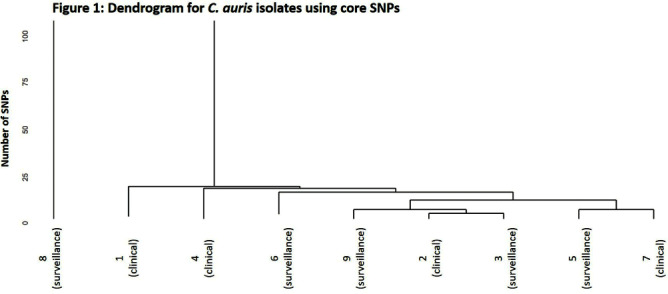# Clinical and Genomic Characteristics of Candida auris in Central Ohio: An Insight into Epidemiological Surveillance

**DOI:** 10.1017/ash.2024.235

**Published:** 2024-09-16

**Authors:** Dhatri Kotekal, Heather Smith, Ryan Carroll, Leama Ajaka, Christina Liscynesky, Amanda Brown, Shashanka Murthy, Christine Sun, Michael Sovic, Shandra Day, Nora Colburn

**Affiliations:** The Ohio State Wexner Medical Center; University of Alabama-Birmingham; The Ohio State University

## Abstract

**Background:** Candida auris is an emerging threat to hospitalized patients and invasive disease is associated with high mortality. This study describes clinical and microbiological characteristics of nine patients identified with C. auris at Ohio State Wexner Medical Center discovered through active surveillance or clinical investigation and uses whole genome sequencing (WGS) to compare isolates. **Methods:** In November 2022, an active C. auris surveillance program was implemented to screen patients admitted to high-risk units (intensive care units and progressive care units). Bilateral axilla and groin swabs were obtained upon unit admission and, if positive, were submitted for C. auris polymerase chain reaction (PCR) with culture and sensitivity testing. Patients with a positive screening or clinical isolate from November 2022 to November 2023 underwent chart review for clinical characteristics, microbiologic data, and index admission information. For each isolate, DNA was extracted and WGS was performed. Core single nucleotide polymorphism (SNP) variation identified from the sequence data was used to infer genetic relationships among the isolates. **Results:** Nine patients were identified between November 2022 and November 2023. The clinical and microbiologic characteristics are summarized in Table 1. All patients were hospitalized at various acute care facilities across the state at least once in the preceding 12 months. C. auris was determined to be present on admission for 6 patients. For 5 of these patients, it was their first interaction with our healthcare system. Three patients were not in contact isolation for >3 days before C. auris was identified. Unit wide point-prevalence screening was completed in these cases and no evidence of transmission was found. WGS showed eight of the nine isolates were related with 28 or less core SNP differences between isolates (Figure 1). One isolate (8) was genetically distinct with >45000 core SNP differences. Five isolates were highly related with a range of 4-15 SNP differences. No temporal or spatial overlap at our institution was identified among these five patients. **Conclusions:** The active surveillance program identified several patients colonized with C. auris in addition to those found through clinical testing. Multiple risk factors for C. auris were identified with high patient mortality (67%). Majority of the isolates were closely related without association with a known outbreak or epidemiologic link, suggesting a possible diffuse common reservoir. Next steps with surveillance in acute care and long-term care facilities will be critical for early detection to halt transmission of this organism.

**Figure:**
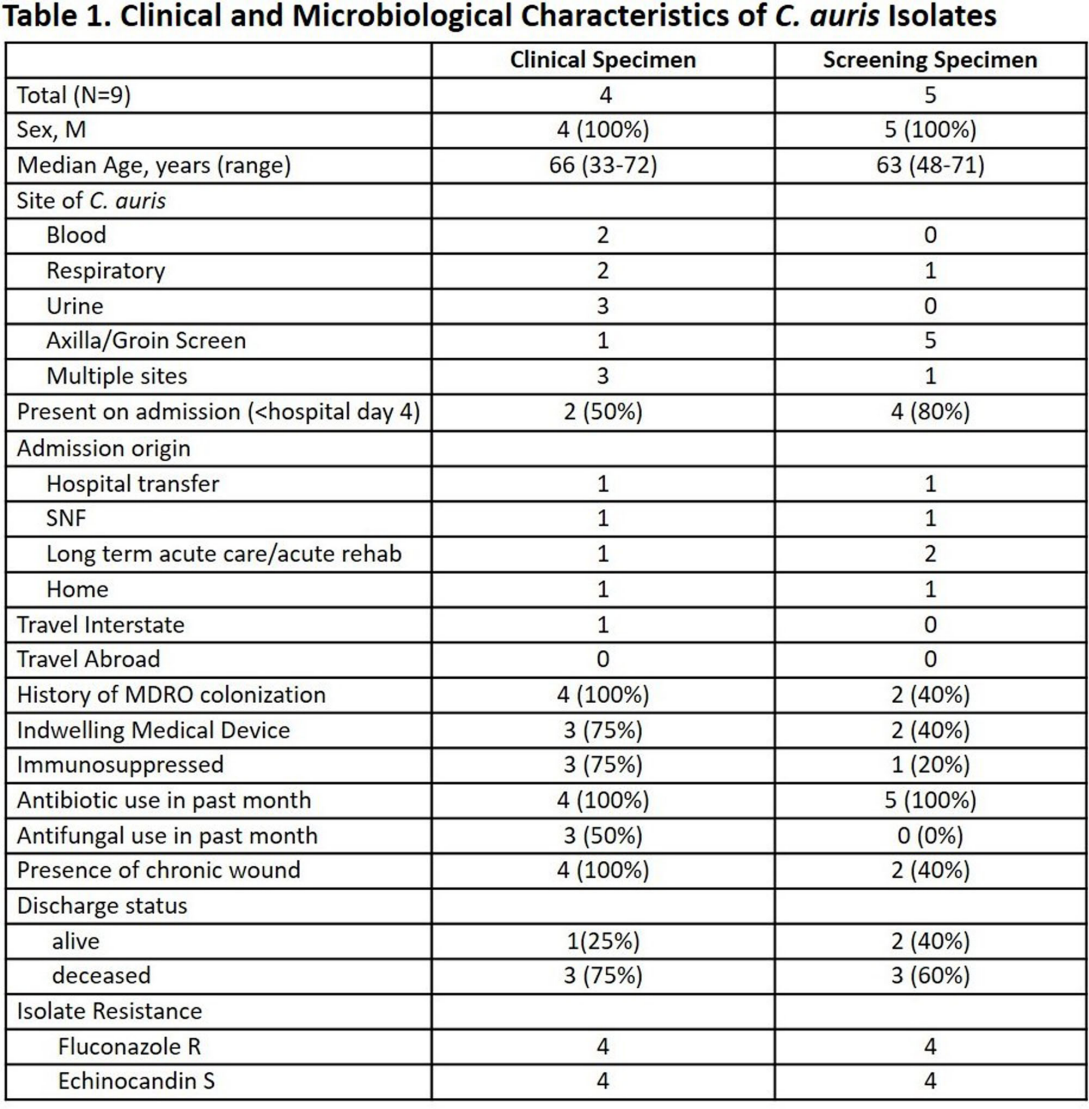

**Figure:**